# Evaluation of the impact of hydrological changes on reservoir water management: A comparative analysis the CanESM5 model and the optimized SWAT-SVR-LSTM

**DOI:** 10.1016/j.heliyon.2024.e37208

**Published:** 2024-09-02

**Authors:** Chenyang Xiao, Mohammad Mohammaditab

**Affiliations:** a College of Resources and Environment, Hubei University of Technology, Wuhan, 430000, Hubei, China; b Sharif University of Technology, Tehran, Iran; c College of Technical Engineering, The Islamic University, Najaf, Iraq

**Keywords:** CanESM5, Shared socioeconomic pathway, Representative concentration pathway, SWAT model, Prediction

## Abstract

This research examines the impacts of climate change and socio-economic variables on the hydrological cycle, reservoir water management, and hydropower capacity at the Gezhouba Dam. The Gezhouba Dam serves as a crucial hydroelectric power station and dam, playing a vital role in regulating river flow and generating electricity. In this study, an innovative method is employed, combining the Soil and Water Assessment Tool (SWAT), Support Vector Regression (SVR), and Long Short-Term Memory (LSTM) models. This model is optimized using the Developed Thermal Exchange Optimizer. This optimized combined model significantly enhances the reliability and precision of the forecasting inflow and reservoir levels. By utilizing the Canadian Earth System Model version 5 (CanESM5), we examine climate variables across various scenarios of Representative Concentration Pathway (RCP) and Shared Socioeconomic Pathway (SSP). Under the SSP5-RCP8.5 scenario, the most aggressive in terms of emissions, we project a temperature rise of 2.6 % and a precipitation decrease of 2.7 %. This scenario leads to the most substantial changes in the hydrological cycle and altered river flow patterns. The results show a direct correlation between precipitation and inflow (0.952) and a strong inverse correlation between temperature and inflow (0.893). The study predicts significant decreases in all flow metrics, with mean high flow (Q5) periods affecting hydropower generation, especially under the SSP5-RCP8.5 scenario. Additionally, the filling frequency rate (FFR) and mean filling level (MFL) are projected to decrease, with a more pronounced decline in the far future, indicating a potential compromise of the reservoir's water storage and power generation capabilities, especially under the SSP5-RCP8.5 scenario.

## 1 Introduction

The planet and humanity are facing one of their most crucial challenges, which is climate change. The impact of climate change can be seen in different areas of the environment and society. This includes the availability of water resources, the generation of energy, ensuring food security, and preserving biodiversity [1]. The changes in the Earth's climate state over an extended period are referred to as climate change, which is primarily the result of human activities that raise greenhouse gas levels in the atmosphere. The water cycle, which involves the storage and motion of water in various locations and forms on Earth, is influenced by climate change [2].

Ecosystems, life, and human actions such as industry, agriculture, and energy production depend on water; therefore, it is a crucial element. Generating electricity using water is called hydropower, which is a form of renewable energy. Typically, hydropower plants depend on reservoirs or dams to store water and regulate its flow through turbines that generate power [3]. Hydropower is the most extensively utilized source of renewable energy, which constitutes approximately 16 % of the world's electricity production [4]. Changes in climate parameters, including temperature, precipitation, and evapotranspiration, have a significant impact on the variability and availability of water resources, which significantly affects the sensitivity of hydropower. Managing and planning hydropower resources effectively requires a comprehensive understanding of how climate change affects their potential and performance [5].

The effects of climate change on hydropower production and water resources can vary according to the location, time of year, and scenario. These impacts can be either negative or positive. This includes the melting of glaciers, alterations in snowmelt patterns, and an increase in extreme precipitation events [6]. These changes are having a significant impact. Water availability for hydropower is negatively affected by the melting of glaciers, although in areas like the Himalayas, it can lead to a growth in water flow [7]. Extreme precipitation can lead to droughts or floods, which can cause significant harm to water storage capability and hydropower infrastructure. Water evaporation and demand are raised by climate change, which results in competition with hydropower and a decrease in its portion of water resources. These parameters threaten the capacity of hydropower to fulfill the world's water requirements [8].

The sectors and regions that rely on hydropower can face noteworthy social, economic, and environmental consequences as a result of these influences. Hence, evaluating the weaknesses and modifications of hydropower to climate change is crucial, and implementing strategies to mitigate the threats and improve the chances is necessary [9].

Assessing the generation of hydropower involves a complex process of predicting climate disparities and analyzing water resources. This task demands the utilization of various scenarios and models. The potential and performance of hydropower plants can be impacted by the variability and availability of water resources, which are affected by climate change. It's crucial to comprehend the consequences of climate change on hydropower since it's a renewable and eco-friendly energy source that relies on the water cycle. This understanding is necessary for effectively managing and planning this essential resource [10].

Accurate prediction of hydroelectric power is vital in ensuring the efficient planning, operation, and management of the electricity system, particularly in raising more renewable energy sources. Hydroelectric prediction involves the utilization of two primary types of models: physical models and data-driven models.

The systems of hydrology and climate are governed by biological procedures that can be mathematically defined in physical models. Statistical analysis of observed or historical data is the basis of data-driven systems, which do not explicitly consider physical procedures. Each approach has its benefits and drawbacks and can be utilized separately or in combination depending on the data accessibility, intricacy, and uncertainty [10].

The classification of hydrological models is according to their spatial resolution and level of detail, which are utilized to demonstrate physical processes that govern the water cycle. Depending on these factors, hydrological models can be categorized into distributed, semi-distributed, or lumped models. Simulating the runoff or streamflow of a river basin is possible. This is the primary input for hydropower generation. By utilizing the forecasted climate variables, it is possible to predict future runoff or streamflow under various climate scenarios [11].

Statistical analysis of observed or historical data is the basis of neural networks, which are another data-driven system [12]. These models can learn from data and adjust to nonlinear and dynamic associations between output and input variables [13]. Moreover, they can simulate the runoff or streamflow of a river basin, as well as predict the future runoff or streamflow under various climate scenarios [14].

Neural networks have several benefits over hydrological models when it comes to hydroelectric prediction [15]. They are capable of handling complicated, noisy, and nonlinear data in hydrological systems, can learn from data without former information of physical procedures or factors, and can provide quick and precise forecasts, which make them valuable for short-term or real-time applications like hydropower operations or flood prediction [16]. Moreover, neural networks can be integrated with other methods or models to enhance their functionality or performance [17].

A thorough review of critical and recent studies conducted on the prediction of hydroelectricity production is presented in this article. Specifically, it focuses on the analysis suggested by Hanoon et al. [14] that hydroelectricity production benefits from its valuable reference. This study investigated the forecast of hydropower production through machine learning algorithms at three Gorges Dams in China. The use of machine learning models has proven to be efficient in predicting variables in various engineering applications. The nature of these variables was highly random, and they could not be identified using traditional mathematical models due to their complexity. So, this investigation was conducted to evaluate the performance of different machine learning algorithms in forecasting the energy output of a dam situated in China, utilizing the information from 1979 to 2016. The study proposed a range of unsupervised and supervised machine learning algorithms, including Auto Regressive Integrated Moving Average (ARIMA), Support Vector Machine (SVM), and Artificial Neural Network (ANN). The study analyzed three distinct scenarios, namely scenario 1 (SC1), which is utilized to forecast daily energy generation; scenario 2 (SC2), which is used to forecast monthly energy generation; and scenario 3 (SC3), which is utilized to forecast seasonal hydropower generation (HPG). Before building the models, the raw data underwent statistical analysis and pre-processing techniques. Five statistical indicators were used to assess the efficacy of the various models developed. The findings showed that the suggested models had the potential to forecast HPG reliably and could be a valuable tool for energy decision-makers. Through sensitivity analyses utilizing graphical distribution data (Taylor diagram), the most suitable models for forecasting HPG for three different scenarios were identified. For the uncertainty analysis, the best models for ANN and SVM were evaluated using 95PPU and d-factors. The outcomes showed that 95PPU values for all models fell within the 80 %–100 % range. Additionally, d-factor values across all scenarios were less than one.

Karakuş et al. [18] employed a deep hybrid CNN-SVR (Convolutional Neural Network-Support Vector Regression) approach utilizing a set of meteorological data as input in order to make forecasts. Between 2007 and 2021, a specific set of data was gathered from the Kaman Meteorology Directorate and Hirfanlı HEPP in Turkey to calculate the daily Hydroelectric Power Generation and Net Head of the HEPP. The benchmarking of the forecast models was performed, including the CNN-SVR model, along with Machine Learning models like Weighted K-Nearest Neighbor Regression (WKNNR)and Boosting Random Forest Regression (BRFR), as well as Deep Learning models such as Deep Belief Network (DBN) and Long-Short Term Memory (LSTM). Upon comparison, the results showed that CNN-SVR outperformed the other models with the maximum correlation coefficient of 0.971 for NH and 0.968 for PP.

Zanial et al. [19] implemented the combination of the Cuckoo search algorithm (CS-ANN) and Artificial Neural Network (ANN). Subsequently, statistical metrics such as Root-Mean-Square Error (RSME) and Determination Coefficient (R2) were used to evaluate and analyze the performance of the algorithm. The Artificial Neural Network (ANN) would be utilized to assess the correctness of the suggested model. The statistical indices demonstrated that the Hybrid CS-ANN model enhanced on the basis of the R2 value in comparison with the Artificial Neural Network model. The R^2^ values obtained during the training and testing phases were 0.900 and 0.935, respectively. During the training stage, the root mean square error (RMSE) value for the Artificial Neural Network model was 127.79 m3/s. In the testing stage, the RMSE value for the Artificial Neural Network model was 12.7 m3/s. On the other hand, the proposed Hybrid CS-ANN model had a root mean square error value of 121.7 m3/s during the training stage and 10.95 m3/s during the testing stage. The findings indicated that the suggested model was better than the separate model in forecasting the river flow with great precision. Even though the proposed model could be employed in diverse case studies, it was essential to adjust the internal parameters of the model when applied in various scenarios.

Wang et al. [20] demonstrated that the causal coupling between them influenced the interdependfigence among subsystems. They have utilized this correlation to suggest a technique for mining time series data and forecasting future trends on the basis of the principles of information causality and the PageRank algorithm. Using a nonlinear model, they were able to demonstrate the effectiveness of their suggested prediction strategy in reducing the auxiliary variables’ dimension. The approach was then tested on a 250 MW hydropower unit, which confirmed that data causal coupling among variables was cross-scale, with definite Markov orders of periods. By considering the information transfer sequences between the prediction object variable and the causal variable in the absence of state auxiliary variables, they were able to improve the accuracy of the forecast. In addition, the suggested technique had the potential to be employed in the exploration of data for other complicated systems and the selection of variables for forecasting models. It effectively linked data-driven and mechanism-based methodologies, which held great significance in engineering applications.

Ehtearm et al. [21] developed a hybrid deep learning method to forecast hydropower generation. Hydropower generation modeling using statistical methods could have been better due to its dependence on periodic and seasonal fluctuations. To achieve more precise predictions, deep learning models could detect daily, weekly, and monthly patterns. The utilization of deep learning models was necessary for forecasting hydropower production because artificial neural networks (ANNs) might not be able to capture nonlinear and latent patterns effectively. In order to extract essential features and forecast results, they employed the Convolutional Neural Network-Multilayer Perceptron-Gaussian Process Regression (CNNE-MUPE-GPRE) model. A binary SSOA was utilized to choose the most fitting input scenarios in the hybrid model. The benefits of this model included the ability to quantify production uncertainty, accurately predict hydropower production, and extract features from input data. The performance of the model was compared to several models, including the Bi-directional LSTM (BI-LSTM), long short-term memory neural network (LSTM), GPRE, MUPE, MUPE-GPRE, CNNE-MUPE, and CNNE-GPRE models. These models were utilized to make predictions on power for the upcoming one, two, and three days. At the 1-day forecast horizon, the CNNE-MUPE, CNNE-MUPE-GPRE, CNNE-GPRE, MUPE-GPRE, BI-LSTM, LSTM, CNNE, GPRE, and MUPE had root mean square error values of 615, 578, 832, 861, 914, 934, 1436,1954, and 1712 KW. For the CNNE-MUPE-GPRE, the RMSE values were 595, 600, and 612 at the 1-day, 2-day, and 3-day forecast horizons. At the first, second, and third days' forecast horizons, the CNNE-MUPE-GPRE model produced RMSE values of 595, 600, and 612, respectively. The accuracy of the model declined as the prediction horizon was extended. Compared to other models, the CNNE-MUPE-GPRE model exhibited lower uncertainty. For more precise hydropower generation forecasts, it was recommended that the CNNE-MUPE-GPRE model be used.

Although previous researchers have used different neural network methods and types of climate models for predictions, the innovation of this study is the use of a Developed Thermal Exchange Optimizer (DTEO) algorithm optimization to increase the accuracy in prediction and the reliability of future conditions.

In this study, we have pioneered the application of the Developed Thermal Exchange Optimizer (DTEO) algorithm, which marks a significant advancement over traditional neural network methods and climate models used for forecasting. The DTEO algorithm stands out for its enhanced predictive accuracy and reliability in simulating future climatic conditions. Despite its strengths, the Thermal Exchange Optimizer (TEO), like many optimization methods, is not immune to the common pitfall of converging to local optima, which can limit its effectiveness.

To address this challenge, our research incorporates cutting-edge modifications that bolster the TEO's exploratory capabilities, thereby mitigating the risk of premature convergence. These enhancements are grounded in the latest expert recommendations and involve sophisticated mathematical alterations to the algorithm's core components. Specifically, we have introduced novel equations that refine the mutation operator and invigorate the local search process within the DTEO framework.

The culmination of these developments is a robust and reliable forecasting tool that sets a new benchmark for predictive modeling in hydrological studies. With the developed TEO algorithm and use it in optimizing the combined hydrological SWAT model with the SVR/LSTM model, dam managers can now forecast with unprecedented precision inflow and water reservoir, enabling them to make well-informed decisions regarding the operation of the Gezhouba Dam under various climatic scenarios. This breakthrough is particularly valuable for adapting to the dynamic and complex patterns of climate change, ensuring the dam's operational efficiency and resilience in the face of environmental uncertainties. In this investigation, the CanESM5 model from the CMIP6 project is utilized, along with different SSP and RCP scenarios, to predict future climate variables as input data optimized SWAT-SVR-LSTM model.

The Gezhouba Dam, located on the Yangtze River, boasts an impressive installed capacity of 2.715 MW and an annual generation of 14,100 GWh. This hydroelectric facility plays a vital role in fulfilling central China's energy requirements and supporting the agricultural and industrial sectors in the region. Nevertheless, the dam's hydropower generation capacity is at risk due to climate change and socio-economic factors. Climate change may lead to glacier depletion, reduced snowmelt, extreme precipitation events, and increased water evaporation, all of which could impact the reliability and quantity of energy produced. Moreover, the growing demand for electricity driven by socio-economic development could strain the dam's ability to meet future energy needs. Despite its strong hydropower generation capacity, the Gezhouba Dam must address challenges posed by climate change and socio-economic growth to ensure its long-term performance and sustainability.

The objective of the study is to evaluate the feasibility of proposed strategies for mitigating and adapting to climate change. This evaluation will take into account various factors such as technological viability, economic cost-effectiveness, regulatory framework, social implications, environmental impact, and carbon footprint. Additionally, the study will consider aspects like potential public acceptance, equity and access, education and awareness campaigns, and ecological implications. These strategies must be designed in a way that does not disproportionately affect specific groups and takes into consideration cultural, social, and behavioral factors. Public education and awareness campaigns will be discussed to encourage the adoption of these strategies. Furthermore, the research will analyze the potential impacts of the strategy on local and regional ecosystems, resource utilization, and the overall effect on greenhouse gas emissions. This comprehensive approach will provide a holistic understanding of the potential impact of these strategies, strengthen policy recommendations, and demonstrate a thorough comprehension of the complexities involved in implementing measures for climate change mitigation and adaptation.

The study is categorized as section 2, which will provide a description of the study area and the sources of data used. Following that, Section 3 will present the methodology and models employed in the study. The results and discussion will be defined in Section 4, while Section 5 will conclude the paper and include recommendations for future research.

## 2 Method and material

### 2.1 Case study description

In the central province of Hubei, the Gezhouba Dam is a hydroelectric power station and an enormous dam on the Yangtze River, which is China's longest river. It is situated in the western suburbs of Yichang city. Yichang, located in the climate zone of a sub-tropical monsoon, experiences four different seasons and maintains an average annual temperature of 15 °C. The primary purpose of its construction was to regulate river flow, produce electricity, and enhance navigation. July and June are the months with the highest temperatures, and during the plum rain seasons, the weather is unsettled, and there are regular rains. Yichang experiences significant amounts of rainfall, with the wettest month being July, which receives 216 mm of rainfall, while December has the lowest levels at 18 mm. The Gezhouba Dam is a reservoir with a capacity of 1.58 Km3, produces 2.71 GW of electricity, and has three ship locks. The central China grid utilizes its power supply to boost agricultural and industrial production in regions including east Sichuan, Hubei, north Hunan, and southwest Henan. The inflow of the reservoir is reliant on the discharge of the Yangtze River upstream, and it receives 451 billion m^3^ of water on average each year. The reservoir can manage the downstream flow by discharging water from its power stations and spillway gates.

### 2.2 Data available

In this investigation, historical information from 2000 to 2024 has been utilized to verify the forecasts made by the hydrological model and climate model. Nine meteorological stations from the NMIC (National Meteorological Information Centre) have collected climatic data, which includes precipitation, temperature, transpiration, evaporation, and wind. Data related to hydrology, precisely the amount of water running off and flowing into the reservoir, have been obtained from six hydrological stations situated along the Yangtze River. These data have been procured from the CMA (China Meteorological Administration). The data regarding the historical electricity production were gathered from the Changjiang Design Group Co., Ltd., which is accountable for managing and operating the Gezhouba Dam. Using these data, a climate/hydrological model was employed to forecast the dam's electricity production from 2024 to 2100.

### 2.3 Climate model description

The CanESM5 (Canadian Earth System Model version 5) is a global model that has been advanced to simulate historical climate change and variability, produce initialized decadal and seasonal forecasts, and create centennial-scale estimates of future climate. The model comprises four parts, namely, the CLASS-CTEM (Canadian land surface scheme and terrestrial biogeochemistry), the CanAm (Canadian atmospheric model), the CMOC and CanOE (Canadian sea ice and ocean biogeochemistry model), and the CanNEMO (Canadian ocean model).

As a global climate model, CanESM5 was developed with the aim of simulating historical climate change and variability, in addition to providing centennial-scale projections of future climate under different SSP and RCP scenarios. In this study, CanESM5 was used to assess changes in important climate variables such as temperature and precipitation. These variables are essential in understanding the potential impacts of climate change on hydrological cycles and, thus, on reservoir inflow and hydropower generation capacity.

Forecasted climate variables by the CanESM5 model under SSPS and RCPs have been utilized as input data optimized SWAT-SVR-LSTM model.

In order to predict the forthcoming climate changes, the model must take into account various scenarios of socio-economic development and greenhouse gas emissions. These particular scenarios are formulated on the basis of the Shared Socioeconomic Pathways (SSPs) and the Representative Concentration Pathways (RCPs). The radiative forcing levels by the year 2100 are described by the RCPs, which refer to the difference between incoming and outgoing energy in the climate of the Earth. The Shared Socioeconomic Pathways represent different pathways of societal development that are on the basis of the challenges associated with climate change adaptation and mitigation.

CanESM5 offers forecasts of temperature and precipitation patterns, which are essential for evaluating changes in the hydrological cycle. In the high-emission SSP5-RCP8.5 scenario, CanESM5 predicts notable temperature rises and modifications in precipitation patterns. The main features of the SSPs and RCPs and their effects on global warming and climate change outcomes are outlined in Table 1.

**Table 1:** The SSPs and RCPs feature.

RCP	SSP	Radiative forcing in 2100 (W/m2)	temperature change in 2100 (°C)	Description and challenges
RCP1.9	SSP1	1.9	1.5	A kind of maintainable pathway restricting warming lower than 1.5 °C is the aspirational purpose of the Paris Agreement. There are challenges for adapting and mitigating.
RCP2.6	SSP1	2.6	1.6	A firm pathway stabilizing warming at 2 °C. There are challenges for adapting and mitigating.
RCP3.4	SSP2	3.4	2.4	An intermediate pathway is stabilizing warming at three °C—medium challenges for mitigating and adapting.
RCP4.5	SSP2	4.5	2.4	An intermediate pathway is stabilizing warming at four °C—medium challenges for mitigating and adapting.
RCP6.0	SSP3	6.0	3.0	A high-emission pathway is stabilizing warming at six °C—high challenges for mitigating and adapting.
RCP7.0	SSP4	7.0	3.8	A high-emission pathway is stabilizing warming at 7 °C. There are high challenges for mitigating and low challenges for adapting.
RCP8.5	SSP5	8.5	4.3	A really high-emission pathway continues to raise warming higher than 8 °C. There are really high challenges for mitigating and low challenges for adapting.

In our study, the CanESM5 model has been integrated with socio-economic variables to provide a comprehensive analysis of potential global futures. The model considers demographic changes, land use changes, economic development, and technological advancements. Population growth, urbanization trends, and age structure are considered, which influence hydropower energy demand, land use patterns, and societal adaptation to climate change. Land use dynamics, such as agricultural expansion, deforestation, and urban sprawl, affect albedo, evapotranspiration rates, and carbon sequestration capacities. Economic development, including growth trajectories and consumption patterns, is used to estimate greenhouse gas emissions. Technological advancements are also considered, considering the potential for solutions to reduce emissions, improve energy efficiency, and enhance climate change mitigation and adaptation efforts. This approach allows for a more nuanced understanding of how socioeconomic pathways can influence climate variables, hydrological cycles, and hydropower potential in the study area.

This investigation examines two potential scenarios for climate change impacts in our study area. The first is the optimistic RCP4.5-SSP1.2, which assumes a low-carbon and sustainable society with a focus on environmental and social equity. The second scenario is the pessimistic RCP8.5-SSP5, which assumes a society heavily reliant on fossil fuels with high consumption levels and limited concern for the environment and social equity. We have compared the results of the CanESM5 model under both scenarios to evaluate the potential range of climate change effects on our investigation area.

### 2.4 Hydrological model description

In this section, an innovative method is employed, combining the Soil and Water Assessment Tool (SWAT), Support Vector Regression (SVR), and Long Short-Term Memory (LSTM) models. This model is optimized using the Developed Thermal Exchange Optimizer. This optimized combined model significantly enhances the reliability and precision of the forecasting inflow and reservoir levels.

The process of integrating the SWAT, SVR, and LSTM models with the DTEO is carried out in a sequential manner, where the output of each model influences the input of the next model. Initially, the SWAT model replicates the hydrological processes and produces runoff data. Subsequently, this data is utilized by the SVR model to conduct regression analysis, capturing nonlinear relationships. The LSTM model, renowned for its capability to retain long-term dependencies, further enhances the accuracy of predictions. The DTEO algorithm optimizes the overall procedure by adjusting the parameters of each model to reduce error metrics such as RMSE while simultaneously maximizing efficiency metrics like the Nash-Sutcliffe Efficiency (NSE).

The DTEO algorithm plays a vital role in the SWAT-SVR-LSTM model, an innovative approach that combines the Soil and Water Assessment Tool (SWAT), Support Vector Regression (SVR), and Long Short-Term Memory (LSTM) networks to predict inflow and water reservoir with exceptional precision.

This algorithm adjusts various parameters in the SWAT model, such as land use, soil characteristics, and topography, to impact inflow. It also fine-tunes the penalty parameter, kernel function, and gamma value in the SVR component, as well as the number of layers, neurons per layer, and learning rate in the LSTM network to improve predictive accuracy. The optimization process focuses on minimizing error metrics while maximizing the Nash-Sutcliffe Efficiency. By utilizing the concept of thermal exchange inspired by Newtonian cooling law, the DTEO algorithm effectively explores the search space, preventing premature convergence to local optima. These enhancements enable the SWAT-SVR-LSTM model to simulate future hydrological scenarios with increased precision and reliability, offering dam managers a powerful tool for strategic water resource planning in the face of changing climatic conditions.

The following subsections are presented in more detail for each of the components used in this innovative method.

#### 2.4.1 The Soil and Water Assessment Tools (SWAT)

SWAT, which stands for Soil and Water Assessment Tool, is a hydrological model that operates on a continuous-time, semi-distributed, and physically based structure. It is designed to forecast the quantity and quality of groundwater and surface water while also evaluating the environmental implications of changes in land usage, climate conditions, and land management practices. The SWAT model plays a significant role in assessing measures to control and prevent soil erosion, manage non-point source pollution and regional management in watersheds. For simulating the inflow into the reservoir through the SWAT model, specific mathematical equations are employed:

The formulation of the water balance for every hydrologic response has been defined as follows [equation (1)]:

(1) $$CapSW_{t}=SW_{0}+\sum_{i=1}^{t}\left(R_{i}-Q_{surf,i}-E_{a,i}-W_{seep,i}-Q_{gw,i}\right)$$

Here, the content of final soil has been represented by $SW_{t}$ (mm), the content of the beginning soil water on the day i has been indicated by $SW_{0}$ (mm), t illustrates the days (time), the precipitation quantity has been indicated by $R_{i}$ (mm), the surface runoff quantity on day i has been illustrated by $Q_{surf,i}$ (mm), the evapotranspiration quantity on day i has been shown by $E_{a,i}$ (mm), the return flow quantity on day i has been indicated by $Q_{gw,i}$ (mm), and the quantity of water entering the zone of vadose from the soil profile on day i has been indicated by $W_{seep,i}$ (mm).

The SCS (Soil Conservation Service) curve number methodology has been utilized in order to estimate the surface runoff [equation (2)]:

(2) $$Q_{surf}=\frac{{\left(R-I_{a}\right)}^{2}}{R-I_{a}+S}$$

Here, the surface runoff has been represented by $Q_{surf}$ (mm), the precipitation has been illustrated by R (mm), the beginning abstraction has been shown by $I_{a}$ (mm), and the possible highest retention has been represented by S (mm).

• The possible highest retention has been computed as [equation (3)]:

(3) $$S=\frac{25400}{CN}-254$$

Here, the curve number has been illustrated by CN that is an application of the type of soil, the condition of antecedent moisture, and usage of land.

• It is assumed that the initial abstraction represents a portion of the maximum possible retention capacity [equation (4)]:

(4) $$I_{a}=\lambda S$$

Here, λ illustrates an empirical coefficient, and often taken into account as 0.2.

• The penman-Monteith methodology has been utilized to calculate the evapotranspiration [equation (5)]:

(5) $$E_{a}=\frac{0.408\Delta \left(R_{n}-G\right)+\gamma \frac{C_{n}}{T+273}u_{2}\left(e_{s}-e_{a}\right)}{\Delta +\gamma \left(1+C_{d}u_{2}\right)}$$

Here, the real evapotranspiration has been represented by $E_{a}$ (mm/day), the slope of the saturation vapor pressure-temperature curve has been shown by Δ (kPa/°C), the net radiation at the crop surface has been demonstrated by Rn (MJ/m^2^/day), the heat flux density of soil has been represented by $G\left({\text{MJ}/m}^{2}/\text{day}\right)$, the constant of psychometric has been demonstrated by γ (kPa/°C), the coefficients $C_{d}$ and $C_{n}$ rely on the stage of time and the kind of reference, the mean air temperature at 2 m height has been represented by T (°C), the speed of wind at 2 m height has been shown by $u_{2}$ (m/s), the actual pressure of vapor has been indicated by $e_{a}$ (kPa), and the saturation pressure of vapor has been demonstrated by $e_{s}$ (kPa).

• The zone of water entering is computed as follows [equation (6)]:

(6) $$W_{seep}=\frac{K_{s}}{\alpha }\left(1-e^{-\alpha \left(SW-FC\right)}\right)$$

Here, the vadose zone of water entering has been represented by $W_{\text{seep}}$ (mm), the conductivity of the saturated hydraulic has been indicated by $K_{s}$ (mm/h), α demonstrates the parameter of a shape (1/mm), the content of soil water has been demonstrated by SW (mm), and the capacity of the region has been demonstrated by FC (mm).

• A conception of a linear reservoir has been utilized in order to compute the return flow [equation (7)]:

(7) $$Q_{\text{gw}}=\frac{\text{GWQMN}}{\alpha_{\text{gw}}}\left(1-e^{-\alpha_{\text{gw}}\text{GW}}\right)$$

Here, the return flow has been indicated by $Q_{\text{gw}}$ (mm), the shallow aquifer's water level must reach GWQMN threshold level before any return flow can occur (mm), the constant of the baseflow recession has been indicated by $\alpha_{\text{gw}}$, and the level of water in the shallow aquifer has been demonstrated by GW (mm).

The SWAT model has been utilized to simulate a reservoir dam in this investigation. Within this model, various equations were employed to simulate the reservoir's hydropower, some of them are provided below:

The formula of the reservoir's water balance has been defined as follows [equation (8)]:

(8) $$ST_{t}=ST_{t-1}+\sum_{i=1}^{n}Q_{in,i,t}-Q_{out,t}-E_{t}-S_{t}$$

Here, the storage of the reservoir at the end of the day t has been indicated by $ST_{t}$ (mm), the storage of the reservoir at the start of the day t has been illustrated by $ST_{t-1}$ (mm), the quantity of subbasins that drain into the reservoir has been demonstrated by n, the inflow from subbasin i on day t has been indicated by $Q_{in,i,t}$ (mm), the reservoir's outflow on day t has been illustrated by $Q_{out,t}$ (mm), the evaporation from the reservoir on day t has been indicated by $E_{t}$ (mm), and the seepage from the reservoir on day t has been demonstrated by $S_{t}$ (mm).

Reservoirs release water based on their specific operating guidelines. These guidelines can be one of the following equations [equations (9), (10), (11)]:

(9) $$Q_{out,t}=Q_{target,t}$$

(10) $$Q_{out,t}=\frac{ST_{t}-ST_{target,t}}{\Delta t}$$

(11) $$Q_{out,t}=\frac{WL_{t}-WL_{target,t}}{A_{t}}\frac{1}{\Delta t}$$

Here, the target release of the day t has been indicated by $Q_{out,t}$ (mm), the target storage of day t has been represented by $ST_{target,t}$ (mm), the level of water on day t has been demonstrated by $WL_{t}$ (m), the target level of water of day t has been illustrated by $WL_{target,t}$ (m), the surface area of the reservoir of day t has been indicated by $A_{t}$ t (m^2^), and the step of time has been illustrated by Δt.

• The evaporation of the reservoir has been computed as follows [equation (12)]:

(12) $$E_{t}=K_{c}ETo_{t}A_{t}$$

Here, the reservoir's evaporation on day t has been represented by $E_{t}$ (mm), open water's crop coefficient has been indicated by $K_{c}$, the reference evapotranspiration of the day t has been indicated by $ETo_{t}A_{t}$ (mm/day), and the surface area of the reservoir has been shown by $A_{t}$ (m^2^).

• The seepage of the reservoir has been computed as follows [equation (13)]:

(13) $$S_{t}=K_{s}A_{t}$$

Here, the reservoir's seepage on day t has been indicated by $S_{t}$ (mm), the coefficient of the seepage has been illustrated by $K_{s}$ (mm/day), the surface area of the reservoir of day t has been represented by $A_{t}$ (m^2^).

Achieving more precise and robust inflow forecasts to a reservoir can be possible by utilizing a combined approach of the SWAT hydrological model and an SVR-LSTM model that is optimized by metaheuristic approaches. This approach enables the temporal, physical, and spatial characteristics of the hydrological system to be considered, as well as the inconsistencies and uncertainties present in both the data and the model. The SWAT-SVR-LSTM model's inflow forecast helps make the reservoir simulation more dependable and accurate by incorporating the current and forthcoming hydrological situations along with the possible effects of climate and land use alteration. An effective strategy to enhance hydroelectric power forecasting and management could be achieved by integrating the SWAT hydrological model with an SVR-LSTM optimized through metaheuristic techniques to forecast reservoir inflow and simulate the reservoir for electricity generation estimation.

#### 2.4.2 The LSTM-SVR model

A combination of support vector machines (SVMs) and long short-term memory (LSTM) networks results in a hybrid model that is referred to as LSTM-SVM. This model is used for various tasks, including sentiment analysis, anomaly recognition, time series forecasts, and signal interference detection. Both extended short-term memory networks and support vector machines have their strengths, and the LSTM-SVM model can capitalize on these advantages. For instance, this model can effectively manage high-dimensional and nonlinear data, as well as model both short-term and long-term dependencies. Furthermore, it can prevent overfitting and avoid local optima [22].

Two major components create the LSTM-SVM model: the SVM classifier and the LSTM network. The LSTM network is a kind of recurrent neural network (RNN) that is capable of processing sequential data and retaining its hidden state over time. A classification of LSTM cells creates the LSTM network, with each cell that is made of an input gate, an output gate, a forget gate, and a cell state [23]. The LSTM network can effectively process input data by learning the feature space and capturing the temporal dependencies of the input data. In a supervised learning algorithm, the SVM classifier is a kind of powerful algorithm that can classify or regress tasks. The SVM classifier can transform the input data into a higher dimension feature space and identify the best hyperplane to separate the data into distinct classes or forecast output values by utilizing a kernel function [24]. The SVM classifier is capable of executing the final organization or forecast task utilizing the document embedding created by the LSTM network as an input piece.

Some of the formulas that the LSTM-SVM model utilizes have been represented as follows:

The updated formula of the LSTM cell state is [equation (14)]:

(14) $$c_{t}=f_{t}⊙c_{t-1}+i_{t}⊙g_{t}$$

Here, the state of cell at the t step of time has been indicated by $c_{t}$, the forget gate has been represented by $f_{t}$, the state of the cell at the t−1 step of time has been illustrated by $c_{t-1}$, the state of the candidate cell has been indicated by $g_{t}$, and the multiplication of the element-wise has been demonstrated by ⊙. it is the input gate output

• The output formula of the LSTM cell output is [equation (15)]:

(15) $$h_{t}=o_{t}⊙\tanh\;\left(c_{t}\right)$$

Here, the output of the cell at the time t has been represented by $h_{t}$, the gate output has been indicated by $o_{t}$, and the function of the hyperbolic tangent has been demonstrated by tanh.

• The formulas of the LSTM gate are [equations (16), (17), (18), (19)]:

(16) $$f_{t}=\sigma \left(W_{f}x_{t}+U_{f}h_{t-1}+b_{f}\right)$$

(17) $$i_{t}=\sigma \left(W_{i}x_{t}+U_{i}h_{t-1}+b_{i}\right)$$

(18) $$o_{t}=\sigma \left(W_{o}x_{t}+U_{o}h_{t-1}+b_{o}\right)$$

(19) $$g_{t}=\tanh\left(W_{g}x_{t}+U_{g}h_{t-1}+b_{g}\right)$$

Here, the vector of the input at the time t has been demonstrated by $x_{t}$, The weight matrices of the input vector include $W_{g}$, $W_{o}$, $W_{i}$, $W_{f}$, the weight matrices of the former cell output include $U_{g}$, $U_{o}$, $U_{i}$, $U_{f}$, the bias vectors include $b_{g}$, $b_{o}$, $b_{i}$, $b_{f}$, and the function of the sigmoid has been illustrated by σ.

• The decision function of the SVM for the organization is [equation (20)]:

(20) $$f\left(x\right)=\text{sign}\left(\sum_{i=1}^{n}\alpha_{i}y_{i}K\left(x_{i},x\right)+b\right)$$

**Fig. 1:**
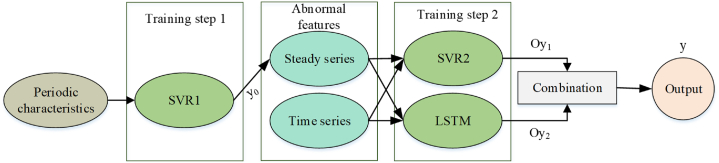
The construction of the SVR-LSTM model.

Here, the input vector has been demonstrated by x, the training samples quantity has been demonstrated by n, the Lagrange multiplier has been illustrated by $\alpha_{i}$, the class label has been indicated by $y_{i}$, the bias term has been represented by b, and the kernel function has been demonstrated by $K\left(x_{i},x\right)$. Fig. (1) shows the construction of the SVR-LSTM model.

**Fig. 2:**
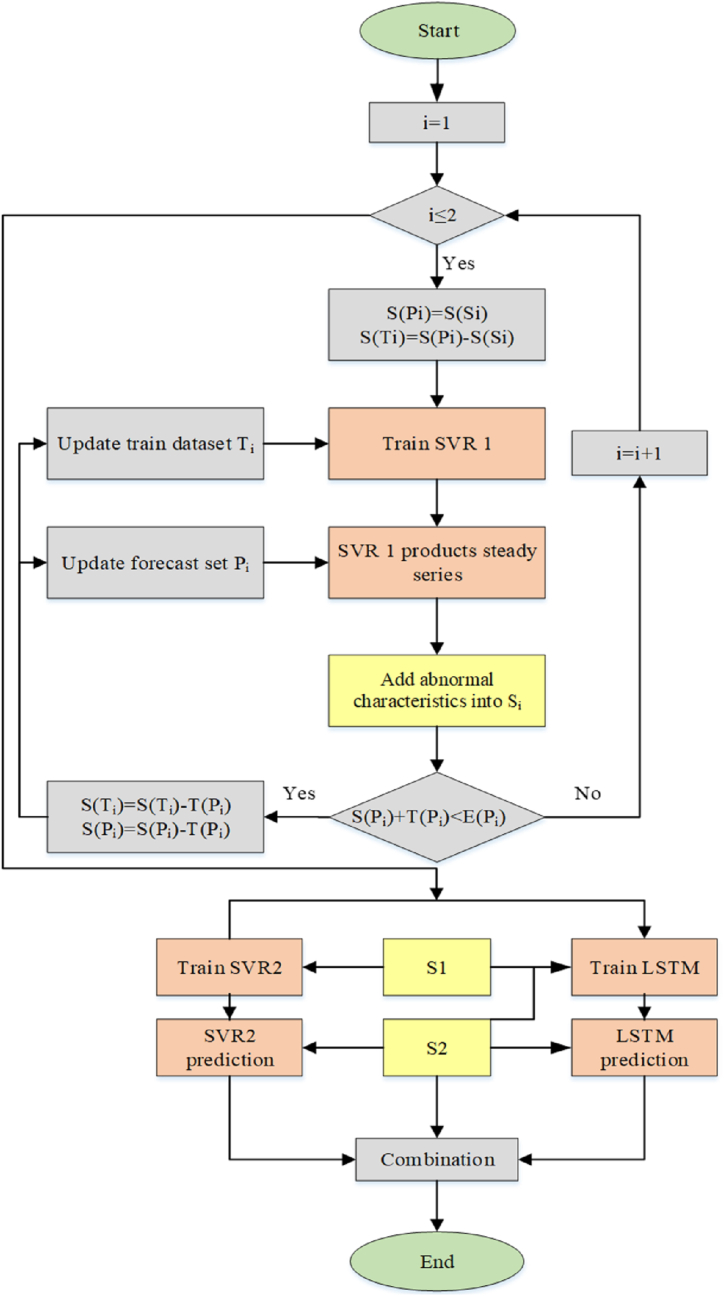
The flowchart for training and forecasting the SVR-LSTM model.

Fig. 2 represents the flowchart for training and predicting the SVR-LSTM. $S_{i}\;\left(i\;\epsilon \;\left\{1,2\right\}\right)$ has been a sample set of input data comprising abnormal characteristics. $T_{i}$ illustrates the sample set utilized for SVR 1 training. $P_{i}$ demonstrates a sample set chosen to compute and gain a steady series for abnormal features in the $S_{i}$. $S_{1}$ is utilized for SVR 2 and LSTM training. $S_{2}$ has also been desired for the LSTM training and the SVR 2 and LSTM-based prediction. The initial times for $T_{i}$, $P_{i}$, and $S_{i}$ are illustrated by $S\left(T_{i}\right)$, $S\left(P_{i}\right)$, and $S(S_{i}$), respectively. The periods for $T_{i}$ and $P_{i}$ are also indicated by $T\left(P_{i}\right)$ and $T\left(T_{i}\right)$, Respectively. $E(S_{i}$) is the end time of $S_{i}$. After creating and planning the construction of the suggested model, the SVR 1 is trained on the basis of the $T_{i}$ and generates a steady series on the basis of $P_{i}$. After that, the abnormal features, such as steady series and time series, are added to $S_{i}$. These procedures are repeated until enough samples for $S_{i}$ are created.

#### 2.4.3 Optimization technique

A. The concept of Newton's law of cooling

The transfer of heat through convection occurs when heat is transferred simultaneously with the movement of fluid. This procedure of heat transfer can be divided into two categories, namely free and compulsory. The presence of natural candidates like the Archimedes principle causes the flow of energy in free motion. Nevertheless, the transportation of fluid takes place due to outside powers, like a mandatory pump or a fan. The evaluation of the heat transfer becomes complicated when fluid movement processes and thermal conductivity are conducted concurrently. The rate of heat transfer increases as the fluid velocity rises. Newton's cooling law expresses the heat transfer's speed as the following formulation [equation (21)]:

(21) $$\bar{H}=\delta \times G\times \left(T_{g}-T_{b}\right)$$

Here, G represents the region that transfers the heat, and the heat is demonstrated by H. Various factors influence the heat transfer process (δ), such as the shape of the object, the texture of its surface, and the mode of heat transfer. Furthermore, $T_{b}$ demonstrates the temperature of the body, and $T_{s}$ illustrates the temperature of the surroundings.

Based on the formula above, the time when heat is lost due to a decrease in temperature (dT) is defined by $\delta \times G\times \left(T_{g}-T\right)\;dt$. This can be represented in the equation below, which describes the change in reserved heat [equation (22)]:

(22) $$V\times \rho \times c\times dT=-\delta \times A\times \left(T-T_{b}\right)dt$$

Here, ρ ($kg/m^{3})$ illustrates the density, $V\left(m^{3}\right)$ demonstrates the volume and $c\left(\frac{\frac{J}{kg}}{K}\right)$ represents the particular heat. As an outcome [equation (23)],

(23) $$\frac{T-T_{b}}{T_{eh}-T_{b}}=e^{\left(\frac{-\delta \times S\times t}{V\times \rho \times c}\right)}$$

Here, λ illustrates a time-autonomous amount and the primary great temperature is demonstrated by $T_{eh}$. [equation (24)]

(24) $$\lambda =\frac{\delta \times S}{V\times \rho \times c}$$

Once again, it is possible to demonstrate the equation above using the following equation [equation (25)]:

(25) $$\frac{T-T_{b}}{T_{eh}-T_{b}}=e^{\left(-\lambda t\right)}$$

Consequently [equation (26)],

(26) $$T=\left(T_{eh}-T_{b}\right)\times e^{\left(-\gamma t\right)}+T_{b}$$

B. Thermal Exchange Optimization Algorithm

The relationship between optimization and the Cooling Law of Newton, as well as the concept of optimization, are discussed in this section. Optimality refers to all methods utilized to achieve the best possible result for optimization problems. Various techniques have been proposed to solve optimization issues. Although conventional methods can solve optimization problems with accuracy, they are less efficient for complicated problems. The utilization of metaheuristic techniques to address various issues and achieve optimal outcomes is highly intelligent. Bio-inspired optimization methods are versatile and can effectively address local optimization problems. Numerous instances from the natural world, such as animal behavior, environmental factors, and upbringing, have been utilized to imitate a process for solving optimal problems through a bio-inspired modeling process. Various types of metaheuristic processes have been proposed recently, such as the ALO (Ant Lion Optimizer) algorithm, EHO (Elephant Herding Optimization), Equilibrium optimizer, WCO (World Cup Optimizer), Biogeography-Based Optimization, and TEO (Thermal Exchange Optimizer).

**Fig. 3:**
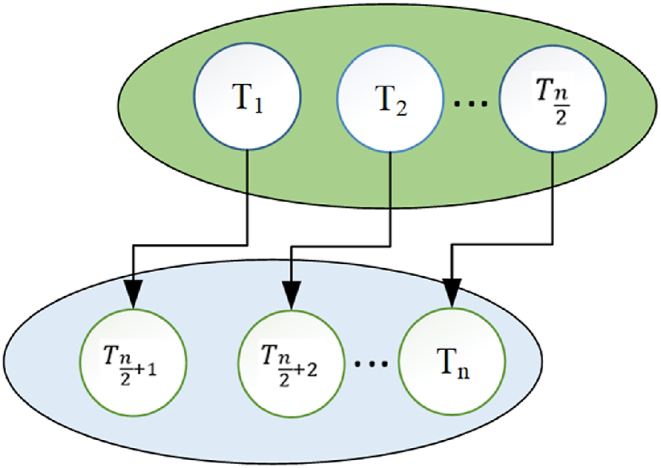
The two different kinds of the transfer.

A more advanced variant of the Thermal Exchange Optimizer method is currently accessible and allows for greater accuracy and dependability in the procedure. The Thermal Exchange Optimizer method includes monitoring the temperature and condition of objects, which are subjected to alternating warm and cold environments to detect any alterations or updates in their state. The candidate in the Thermal Exchange Optimizer has been divided into two groups for the experiment. One group was tasked with cooling materials, while the other group was responsible for regulating the environment. The process was then reversed. Two different types of transfer are depicted in Fig. (3).

To begin the procedure, a set of random individuals previously defined as initial outcomes were utilized. These individuals have been represented as follows [equation (27)]:

(27) $$T_{j}^{0}=\underline{T}+\xi \times \left(\bar{T}-\underline{T}\right)\;i=1,2,\ldots ,n$$

Here, the value ξ stands for a random number that ranges from 0 to 1. The value $T_{i}^{0}$ illustrates the main population of the process for the item i. The value $\bar{T}$ indicates the maximum boundary, and $\underline{T}$ represents the minimum boundary. First, the cost function of the created candidates has been obtained, and then the Thermal Memory (TM) saves the top-cost individuals that are represented by T in order to offer more excellent production by using fewer problems in the process.

Initially, the TM members are combined with the current members. Subsequently, an equal count of the most undesirable members is eliminated. $T_{\frac{n}{2}+1}$ cooling item's surrounding item has been expressed by $T_{1}$, and on the other hand, $T_{\frac{n}{2}+1}$ expresses the surrounding item for $T_{1}$. If the element is less than λ, the temperature alters slowly. The value λ has been computed through the below equation [equation (28)]:

(28) $$\lambda =\frac{\mathrm{Cos}\;\left(item\right)}{\mathrm{Cos}\;\left(the\;worst\;item\right)}$$

In order to accurately imitate the optimization algorithm, the time has been taken into account as an additional duration that is associated with the number of repetitions performed. This period has been computed utilizing the below formula [equation (29)]:

(29) $$t=\frac{repetitions\;}{\mathrm{Max}.repetitions\;}$$

Enhancing the exploration of a procedure has been attributed to the temperature changes in the environment, which is computed by the below formula [equation (30)]:

(30) $$T_{j}^{l}=\left(1-\left(\alpha_{1}+\alpha_{2}\times \left(1-t\right)\times \varphi \right)\right)\times {T_{j}^{\prime }}^{l}$$

Here, φ is a stochastic amount that ranges between 0 and 1. The value ${T_{j}^{\prime }}^{l}$ indicates the temperature of the element that $T_{i}^{l}$ completes it. The values $\alpha_{1}$ and $\alpha_{2}$ are control factors.

At last, the novel position of the element's temperature is obtained by the below equation [equation (31)]:

(31) $$T_{j}^{N}=T_{j}^{l}+\left(T_{ij}^{old}-T_{j}^{l}\right)e^{\left(-\varphi t\right)}$$

Furthermore, this process clarifies whether a component alters the cooling's objects or not. It is iterated by a duration that is indicated by Pr that involves several members contrasted with R(i), which is a stochastic number in a limited region of 0–1. One of the possible member's dimensions is stochastically selected if the value R(J) is smaller than Pr. The quantity of $T_{j,i}$ is computed by the below formula [equation (32)]:

(32) $$T_{j,i}={\underline{T}}_{i}+\sigma \left({\bar{T}}_{i}-{\underline{T}}_{i}\right)e^{\left(-\varphi t\right)}$$

Here, $T_{j,i}$ illustrates the $j^{th}$ parameter amount of the $i^{th}$ population. The highest boundary of the variable i is indicated by ${\bar{T}}_{j}$, and the lowest boundary of the variable i is illustrated by ${\underline{T}}_{j}$. The process ends when the criteria values are fulfilled.

C. Developed Thermal Exchange Optimizer (DTEO)

Identifying the best global solution is among the numerous advantages of the Thermal Exchange Optimizer (TEO) algorithm. However, it demands attention since it also has certain shortcomings. It is quite easy for the algorithm to get trapped in local optima, which is a crucial shortage. Many experts have suggested various modifications to enhance the exploration abilities of the metaheuristics to overcome this limitation. In this specific investigation, our study involves an enhancement procedure that aims to improve the Thermal Exchange Optimizer Algorithm. Mathematical modifications to the Thermal Exchange Optimizer algorithm can be achieved by introducing equations that will apply to the mutation operator and local search process. Here are the enhanced formulas.

Algorithm 1 Developed Thermal Exchange Optimizer (DTEO)

**Table:** 

1. Start the stochastic members utilizing equations. 33 and 34 as beforehand. 2. Assess the performance index for every member and save the situations of the top-cost candidates in the Thermal Memory (TM). 3. Appeal the Thermal Exchange procedure as explained in the original algorithm. 4. Present a mutation operator that stochastically disturbs a subset of the member solutions. This can be completed by including a mutation term to the former temperature computation in equation. (35): $T_{j}^{N}=T_{j}^{l}+\left(T_{ij}^{old}-T_{j}^{l}\right)e^{\left(-\varphi t\right)}+\varepsilon$ (33) Where ε is a stochastic mutation term expressed as: $\varepsilon =\mu \times \left(\bar{T}-\underline{T}\right)$ (34) And μ is a stochastic value that ranges from 0 to 1. 5. Implement a local search around the finest member in the TM. This includes perturbing the member solution and assessing their enhanced fitness. Let's represent the temperature of the finest member in the TM as $T_{best}$. The local search operation can be expressed as the following formula: $T_{local}=T_{best}+\delta \times \left(\bar{T}-\underline{T}\right)$ (35) Here, δ represents a stochastic value that ranges from 0 to 1. 6. Assess the performance index for the mutated members from the mutation operator and the local search member $T_{local}$. Update the TM if essential. 7. Reiterate phases 3 to 6 for a pre-expressed amount of iterations or until a stopping criterion is fulfilled (e.g., getting the highest amount of function assessments). 8. Return the finest solution found in the TM.

By including a mutation term in the temperature computation, these mathematical changes enable a more comprehensive exploration of the search space. In addition, the exploitation operation disturbs the most favorable member present within the TM, with the aim of improving the search outcomes in the immediate area. These enhancements increase the algorithm's ability to explore and exploit the search space, which can lead to better optimization results.

D. Validation of the algorithm

An evaluation was carried out on established benchmark functions from the CEC-BC-2017 test case to assess the efficacy of the Developed Thermal Exchange Optimizer (DTEO). Six new distinct optimization methods, Sun Flower Optimization (SFO) [25], Lion Optimization Algorithm (LOA) [26], World Cup Optimization algorithm (WCO) [27], Manta Ray Foraging Optimization (MRFO) [28], Trimet Graph Optimization (TGO) [29], Spotted Hyena Optimizer (SHO) [30] were used to compare the obtained results. A comparison was made between the results of the Developed Thermal Exchange Optimizer (DTEO) and those obtained using established optimization methods in order to assess its competitiveness and efficiency.

### 2.5 The hydropower potential

Utilizing water to generate electricity or power machines is known as hydropower potential. It is a renewable and eco-friendly energy source that doesn't emit any greenhouse gases or harmful substances. Additionally, it offers advantages such as flood mitigation, irrigation, recreation, and water usage. Converting mechanical and electrical energy from moving water is the fundamental concept of hydropower generation. The power generated is directly proportional to the ratio of flow and head of the water, with increased flow rates producing more power. Hydroelectric dams are the most commonly used method to generate hydropower. These dams block rivers and make a reservoir. The water from the reservoir is then released over a penstock and flows through a turbine. The turbine is attached to a generator which converts the rotational motion of the turbine into electrical current. The electrical current is then sent to the consumers or power grid.

The theoretical power accessible from hydropower has been mathematically defined as follows [equation (36)]:

(36) P=ρqghη

Here, the power has been illustrated by P (W), the water density has been indicated by ρ (kg/m3), the ratio of flow has been illustrated by q (m3), the gravity's acceleration has been demonstrated by g (m/s^3^), h indicates the head (m), and the system's efficacy has been represented by η that often ranges between 0.7 and 0.9.

### 2.6 Metrics for evaluating and quantifying effects

The purpose of this study is to assess how the suggested dam will perform considering the impact of climate change. The study examines 11 different factors to measure and assess the effects of climate change on the dam's reservoir, including changes in upstream water flow, how the reservoir fills up, and the characteristics of hydroelectric power potential.

The storage and power potential of a reservoir relies heavily on the natural flow upstream, while the total runoff sets the maximum limit for available water resources. The efficacy and performance of the reservoir are significantly affected by the storage during the flood season and the discharge levels that are either too high or too low. Two future times are being evaluated to determine the gross amount of flow and alterations in hydrological extremes under climate change scenarios. The Mean Annual Flow (MAF) represents the average quantity of water that passes through a river on a yearly basis. On the other hand, the Mean Flood Season Flow (MMF) refers to the average amount of water that flows through a river during the flood season, which typically occurs in the wettest month of the year. Q5, also known as high flow, represents the upper portion of the flow distribution, indicating the flow rate that exceeds 5 % of the time. Conversely, Q95, referred to as low flow, represents the lower portion of the flow distribution, indicating a flow rate that exceeds 95 % of the time.

Ensuring the dependability of the entire storage system during the period of flood recession, as well as maintaining an appropriate storage level, are crucial factors in determining the advantages that can be obtained from the proposed dam. To investigate the effects of climate change on reservoir dewatering efficacy for two distinct periods, three criteria have been chosen: Mean First Full Fill Day (MFD), filling rate (FFR), and Mean Fill Level (MFL).

The main focus of hydropower facilities is on the total amount of electricity generated, and fluctuations from year to year can have a significant effect on the reliability of the power supply. The efficiency of water resource usage for electricity production can be measured by the mean annual power (MAP), the coefficient of variation in power output across years (CV), the guaranteed power generation rate (PAR), and the rate of water that is spilled (SPW).

### 2.7 Statistical performance metrics

Statistical performance metrics, including Mean Square Error (MSE), correlation coefficient (R), Mean Absolute Error (MAE), Root Mean Square Error (RMSE), and Mean Absolute Percentage Error (MAPE), are presented and employed to evaluate the outcomes in this article. The calculations for these metrics are [equation 37–41]:

(37) $$R=\frac{\sum_{l=1}^{N}{\left(x_{l}-\bar{x}\right)}^{2}\left(y_{t}-\bar{y}\right)}{\sqrt{\sum_{l=1}^{N}{\left(x_{l}-\bar{x}\right)}^{2}\sum_{l=1}^{N}{\left(y_{l}-\bar{y}\right)}^{2}}}$$

(38) $$MSE=\frac{\sum_{l=1}^{N}{\left(x_{l}-y_{l}\right)}^{2}}{N}$$

(39) $$RMSE=\sqrt{\frac{\sum_{l=1}^{N}{\left(x_{l}-y_{l}\right)}^{2}}{N}}$$

(40) $$MAE=\frac{\sum_{l=1}^{N}\left|\left(x_{l}-y_{l}\right)\right|}{N}$$

(41) $$MAPE=\frac{\sum_{l=1}^{N}\frac{\left|\left(x_{l}-y_{l}\right)\right|}{x_{l}}}{N}\times 100$$

Every metric has a unique way of evaluating the results. The correlation coefficient between the actual and predicted values of the model is depicted by R. The metric MSE represents the mean of the squares of the errors, which is the mean squared division between actual and predicted values of the model. RMSE, on the other hand, is a quadratic error metric that shows the standard deviation of errors. The mean difference between the predicted and actual values is determined by MAE. The metric MAPE is often employed in regression and time-series problems to dassessthe precision of predictions. For evaluating results using such metrics, the ideal state is the highest R value and the lowest values for predictive error evaluation metrics.

## 3 Results and discussion

### 3.1 The forecasting climate variable

The projected alterations in climatic variables have been produced using a model utilizing CMIP6, encompassing six socio-economic and climate scenarios. An analytical and forecasting approach has been used to formulate these projections, using historical data from 2000 to 2023. The detailed presentation of the resultant simulation for different climatic variables may be seen in Table 2.

**Table 2:** Forecasting climatic variables for both distant and proximate periods.

		Near future (2024–2050)			Far future (2051–2100)		
Climate parameters	actual	SSP1-RCP2.6	SSP2-RCP4.5	SSP5-RCP8.5	SSP1-RCP2.6	SSP2-RCP4.5	SSP5-RCP8.5
Precipitation (mm)	1285	1273	1268	1257	1270	1259	1245
Temperature (°C)	16.52	16.66	16.81	16.95	16.71	16.95	17.12
Wind (m/s)	2.44	2.46	2.48	2.5	2.47	2.5	2.53
Evaporation (mm)	2.52	2.54	2.56	2.6	2.56	2.58	2.61

The simulation results indicate a steady increase in temperature, with projections suggesting a rise of 1.4 % under the SSP1-RCP2.6 scenario, 1.7 % under the SSP2-RCP4.5 scenario, and 2.6 % under the SSP5-RCP8.5 scenario. This consistent increase is expected to continue in the future. However, there is a significant decline in precipitation patterns, with predicted decreases of 0.93 %, 1.3 %, and 2.7 % under the SSP1-RCP2.6, SSP2-RCP4.5, and SSP5-RCP8.5 scenarios. These reductions could affect local water supplies and ecological systems.

Wind velocity is also predicted to rise, with forecasts predicting a rise of 0.81 %, 1.6 %, and 2.4 % under the SSP1-RCP2.6, SSP2-RCP4.5, and SSP5-RCP8.5 scenarios. This could affect industries like energy production and agriculture, which are susceptible to wind conditions. Additionally, evaporation rates are expected to rise by 3.97 % under the SSP1-RCP2.6 scenario, 7.54 % under the SSP2-RCP4.5 scenario, and 11.11 % under the SSP5-RCP8.5 scenario. In anticipation of future developments, evaporation rates are projected to increase by 6.75 % under the SSP1-RCP2.6 scenario, 9.13 % under the SSP2-RCP4.5 scenario, and 12.3 % under the SSP5-RCP8.5 scenario. These projections highlight the potential impact of climate change on various sectors, including water supply and ecosystem dynamics.

### 3.2 SWAT-LSTM-SVR performance evaluation

In this section The Developed Thermal Exchange Optimizer (DTEO) is thoroughly evaluated using established benchmark functions from the CEC-BC-2017 test case. The evaluation involved comparing the results with six other state-of-the-art optimization approaches: Sun Flower Optimization (SFO) [31], Lion Optimization Algorithm (LOA) [32], World Cup Optimization algorithm (WCO) [33], Manta Ray Foraging Optimization (MRFO) [28], Trimet Graph Optimization (TGO) [29], Spotted Hyena Optimizer (SHO) [34]. To ensure a fair comparison, consistent parameter settings were used across all algorithms, with a maximum of 200 epochs and a population size of 50. The evaluation was conducted separately for each method, with 30 iterations performed across all benchmark functions to ensure reliable and accurate results. A comparative analysis was then carried out to assess the efficacy and competitiveness of the DTEO algorithm against known optimization strategies.

The research employs functions that have a solution range of −100 to 100. Ten dimensions distinguish each function. The evaluation results, which compare the ATEO algorithm with other metaheuristic algorithms on the CEC-BC-2017 test functions, are presented in Table 3.

**Table 3:** The DTEO optimizer with other metaheuristic optimizers on the CEC-BC-2017.

Function	Indicator	DTEO	SFO	LOA	WCO	MRFO	TGO	SHO
F1	Avg	5.425	23.16	36.26	53.87	27.64	8.73	81.73
	StD	3.692	18.71	26.36	28.48	33.34	4.38	0.00
F3	Avg	8.616	186.69	23.91	13.73	8.92	165.59	151.93
	StD	2.372	0.00	8.03	8.67	850.07	0.00	0.00
F5	Avg	187.291	341.24	460.87	397.32	3320.26	239.46	235.93
	StD	13.795	3.15	4.23	2.66	29.14	33.51	26.77
F7	Avg	52.417	297.94	362.77	386.75	537.30	235.11	228.31
	StD	4.391	4.80	0.82	1.12	1.83	1.30	1.27
F9	Avg	289.141	344.84	473.02	459.15	425.48	295.48	319.36
	StD	2.221	2.47	2.65	4.97	11.61	4.24	3.86
F11	Avg	381.159	630.73	569.81	724.46	795.29	566.63	555.90
	StD	4.411	4.98	5.71	6.94	15.06	6.86	7.37
F13	Avg	4591.598	5122.74	1235.72	11567.85	25750.18	4248.43	5248.11
	StD	2061.395	3816.57	2930.23	4660.34	3104.96	70.30	61.62
F15	Avg	156.222	760.63	1126.41	1939.38	5819.37	1024.52	1039.31
	StD	48.343	59.10	64.72	788.97	4758.64	321.04	311.32
F17	Avg	713.187	951.36	761.50	789.91	1400.82	785.42	691.72
	StD	28.06	17.20	20.82	28.39	35.62	44.47	40.18
F19	Avg	705.568	1525.91	1252.47	2324.09	761.32	784.56	725.79
	StD	44.637	804.47	987.62	4040.28	1803.16	19.68	19.77

The DTEO optimizer outperformed six other metaheuristic optimizers on the CEC-BC-2017 benchmark functions, with a lower average value and standard deviation indicating superior performance. The DTEO optimizer beat the other optimizers on 8 out of ten functions and displayed the lowest StD values on most functions, indicating high reliability and consistency. It also outperformed the other optimizers significantly on challenging functions like F13, F15, and F19.

The DTEO optimizer has several advantages over other algorithms, including a dynamic adjustment strategy that balances exploration and exploitation abilities and a local search operator that refines solutions and improves accuracy. This feature enables the DTEO optimizer to achieve high-quality solutions quickly.

The algorithm's efficacy is demonstrated in optimizing the LSTM-SVR and the SWAT hydrological model to facilitate precise inflow and reservoir storage simulation, particularly within the power generation domain. The study begins with the simulation of incoming flow and then analyzes the tank's storage capacity.

The study employed the optimized SWAT-LSTM-SVR model to assess the impact of climate change on inflow. The model utilized forecasted climate data as input. The calibration phase involved using 70 % of the input data for simulation and reserving the remaining 30 % for validation. Monthly natural streamflow data from 2000 to 2017 was used for calibration, while data from 2018 to 2023 was used for evaluation. After calibration, the model was executed for each climate scenario under different Representative Concentration Pathways (RCPs). The performance of the calibrated SWAT-LSTM-SVR model was evaluated using the Nash-Sutcliffe efficiency (NSE), Root Mean Square Error (RMSE), and coefficient of determination (R2). A value exceeding 0.5 was deemed acceptable. The simulation accuracy of the optimized SWAT-LSTM-SVR model is presented in Table 4.

**Table 4:** The simulation accuracy of optimized SWAT-LSTM-SVR.

Metrics	Calibration	Validation
R^2^	0.91	0.83
NSE	0.79	0.71
RMSE	0.29	0.48

Based on the data presented in Table 4, the simulation's overall accuracy is deemed favorable. The Nash-Sutcliffe Efficiency (NSE) values range from 0.79 during the calibration period and 0.81 to 0.71 during the validation period, indicating a consistent level of accuracy. The Root Mean Square Error (RMSE) values range from 0.29 during the calibration and 0.48 during the validation period, indicating a consistent level of accuracy. The R^2^ values also demonstrate a high level of accuracy, with a range of 0.91 during the calibration phase and 0.83 during the validation phase.

Notably, the calibration and validation period were the same. These findings highlight the strong performance of the SWAT-LSTM-SVR model in accurately representing the variations in real streamflow under different climatic conditions. The results indicate that the model performed well throughout the calibration period, with a close agreement between observed and simulated data. This time frame exhibits the strongest association between the actual and simulated data, suggesting that the model captures runoff dynamics across different climatic scenarios well.

The integration of the DTEO optimizer into the SWAT-LSTM-SVR model signifies a notable progression in hydrological simulation, especially when compared to previous studies—for example, Shaker et al. [35] utilized machine learning techniques like XGBoost and SVR in conjunction with distributed hydrological models such as SWAT and SWAT_Glacier1. Their research demonstrated that the LSTM model achieved NSE and R2 values surpassing 0.80 during calibration and validation.

Likewise, Ji et al. [36] applied LSTM to replicate daily discharge in data-scarce glaciated watersheds in the Tianshan Mountains, Central Asia2. Their outcomes were encouraging, as the LSTM simulations closely matched observations, with NSE and R2 values surpassing 0.70.

These investigations highlight the potential of LSTM in hydrological modeling. Nevertheless, the innovative utilization of the DTEO optimizer to enhance the SWAT-LSTM-SVR model is particularly remarkable for its untapped capacity to enhance simulation precision and effectiveness. This inventive approach may lead to more accurate and dependable hydrological forecasts, especially in the face of climate change and its implications for water resource management.

### 3.3 The inflow simulation

**Fig. 4:**
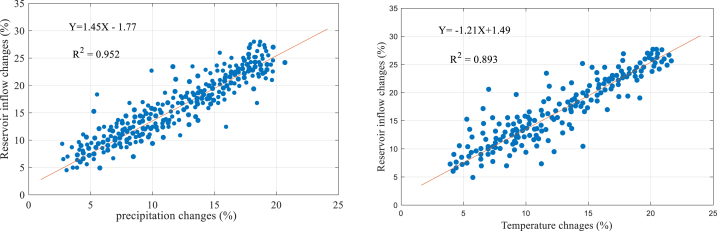
The correlation between climate variable with reservoir inflow.

Also, the correlation between the simulated precipitation and temperature data has been evaluated with the inflow to reservoir. Fig. (4) shows inflow response to rainfall and temperature changes.

According to the findings, the data show a direct linear relationship between precipitation and the flow rate entering the reservoir. The correlation between rainfall and flow shows a robust direct correlation (R^2^ = 0.952), while there is a strong inverse correlation between temperature data and flow (R^2^ = 0.893). Further analysis shows that for every 10 % decrease in rainfall, the simulated mean annual inflow decreases by 12.73 %. However, the effect of temperature increase on current response could be more substantial, as a 10 % increase in temperature only leads to a 10.61 % decrease in inflow current.

**Fig. 5:**
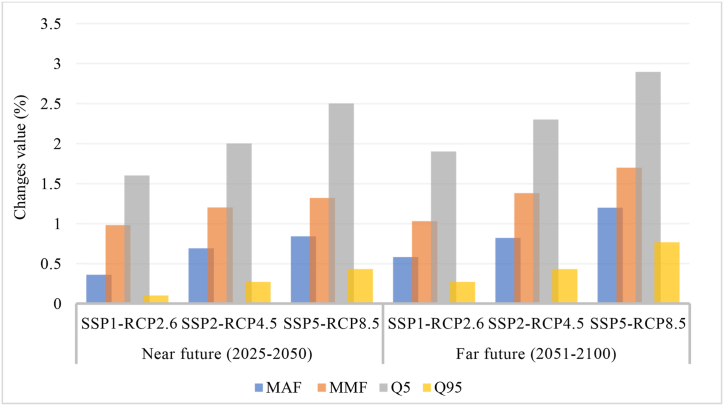
Forecasting changes in the ranges of MAF, MFF, Q5, and Q95 by SWAT-under SSPs-RCPs.

Fig. (5). Illustrates the Forecasting changes in the ranges of MAF, MFF, Q5, and Q95 by LSTM -SVR-SWAT-DTEO under scenarios. According to the research, the predicted changes for Mean Annual Flow (MAF), Mean Flood Season Flow (MFF), Mean High Flow (Q5), and mean low flow (Q95) are on the decrease, with a more substantial reduction predicted in the far future. Rapid economic expansion and the use of fossil fuels are features of the SSP5-RCP8.5 scenario, which raises greenhouse gas concentrations and radiative forcing, causing more warming and modifications to the hydrological cycle. With its emphasis on development and equality, the SSP1-RCP2.6 scenario protects the global commons and honors nature's bounds, which leads to less warming and modifications to the hydrological cycle. With the most significant percentage fluctuations, the Q5 measure indicates greater variability under high flow conditions. The Q95 metric changes less across defined times than another inflow group, according to the mean yearly flow and flood season flow, which exhibit more considerable percentage fluctuations.

The percentage fluctuations forecasting under SSP1-RCP2.6 with a MAF of 0.4 %, MFF of 0.9 %, Q5 of 1.6 %, and Q95 of 0.3 %, and under SSP2-RCP4.5 with a MAF of 0.7 %, MFF of 1.1 %, Q5 of 2 %, Q95 0.3 %, and under SSP5-RCP8.5 with a MAF of 0.9 %., MFF is about 1.5 %, Q5 of 2.5 %, and Q95 of 0.4 % by 2050. Also, the inflow forecasting under SSP1-RCP2.6 with a MAF of 0.6 %, MFF of 1 %, Q5 of 1.8 %, and Q95 of 0.4 %, and under SSP2-RCP4.5 with a MAF of 0.8 %, MFF of 1.3 %, Q5 of 2.3 %, Q95 0.4 %, and under SSP5-RCP8.5 with a MAF of 1.1 %., MFF at about 1.6 %, Q5 at 2.8 %, and Q95 at 0.8 % by 2100.

According to the obtained values, the fluctuations by 2100 are higher than by 2050, and under the SSP5-RCP8.5 scenario, it is higher than other scenarios.

**Fig. 6:**
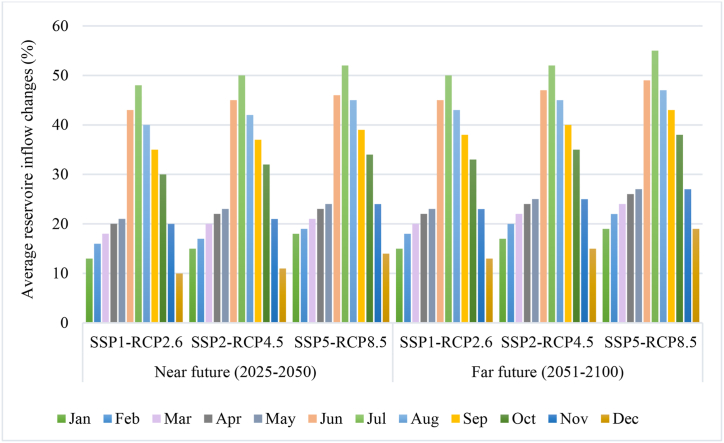
The changes in average monthly inflow.

Fig. (6) illustrates the changes in average monthly inflow to the reservoir compared to the reference period considering socioeconomic and climate scenarios for the timeframes of 2023–2050 and 2051–2100. The projected average inflow for different months in both future periods indicates a general decrease. Specifically, there is a notable decline in quantity during the dry months, particularly in January and February. Conversely, the most significant reductions are observed during the flood season, specifically in June and July. Also, the magnitude of changes in the distant future is more severe than the changes predicted in the near future.

The reservoir's average monthly inflow is projected to experience a significant increase by 2050 under the SSP5-RCP8.5 scenario, with a minimum reduction of 10 % in June. Looking ahead to 2100, the maximum increase is anticipated to be slightly below 60 %, while the minimum decline is estimated to be around 40 %. These projections, which rely on existing climate models and scenarios, play a vital role in informing future water resource management strategies.

### 3.4 The reservoir storage simulation

**Fig. 7:**
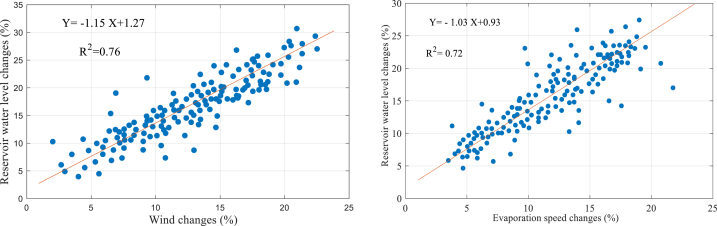
Correlation between wind speed and evaporation with reservoir water level.

This section evaluates the relationship between wind speed parameters and evaporation with the reservoir water level. Fig. (7) shows the response of the water level to changes in wind speed and evaporation in the future.

The data analysis reveals a direct and proportional relationship between wind speed and evaporation with the reservoir's water level. This correlation is supported by correlation coefficients (R^2^ = 0.76) and (R^2^ = 0.72), indicating a significant impact of these climatic factors on the reservoir water level.

Specifically, for every 10 % increase in wind speed, the average water level of the reservoir decreases by 10.23 %. On the other hand, the response of tank storage to evaporation is not as pronounced, as a 10 % increase in temperature only results in a 9.37 % decrease in the reservoir surface area.

**Fig. 8:**
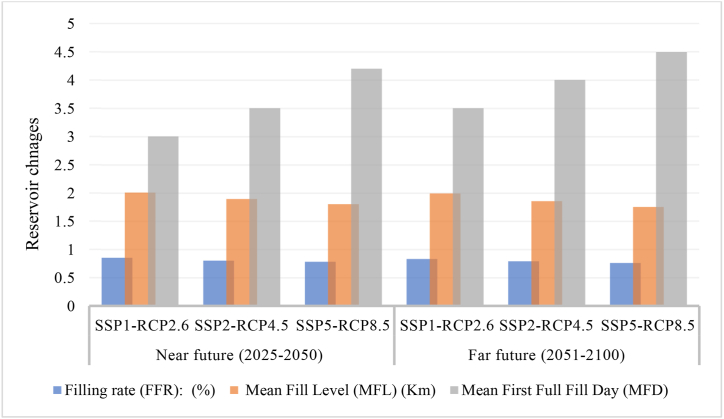
The reservoir changes under climate and socio-economic scenarios.

The alterations in the range of reservoir storage criteria in comparison to the reference period under various SSPs-RCPs are illustrated in Fig. (8).

The study shows that higher SSPs-RCPs decrease FFR values, indicating lower reservoir filling rates. The distant future values are higher than the near future, implying a decrease in water input due to reduced rainfall and runoff. The near future has the highest FFR value of 0.85 %, while the far future has the lowest at 0.76 %.

The MFL values correlate negatively with RCP8.5-SSP5 scenarios, suggesting reduced reservoir filling due to increased emissions. The far-off future values are lower than near-future values, meaning a decline in reservoir water storage capacity due to evaporation and reduced rainfall. The near future has a maximum value of 2.011 m, while the far future has a minimum value of 1.752 m.

The MFD values show an upward trend in scenarios with RCP8.5-SSP5 scenarios, indicating increased emissions leading to higher averages on the initial day of reservoir filling. These values are even higher in the distant future, suggesting a longer duration for the reservoir to reach total capacity due to reduced inflow and heightened outflow. The minimum value is three days for the near future and 4.5 days for the far future.

The results demonstrate that climate change exerts a noteworthy influence on filling quantities, average filling levels, and the initial day of complete filling for tanks. Furthermore, these effects vary depending on the degree of greenhouse gas emissions and socio-economic development. The findings also demonstrate the need for mitigation and adaptation measures to enhance reservoir water management and guarantee long-term sustainability and dependability.

**Fig. 9:**
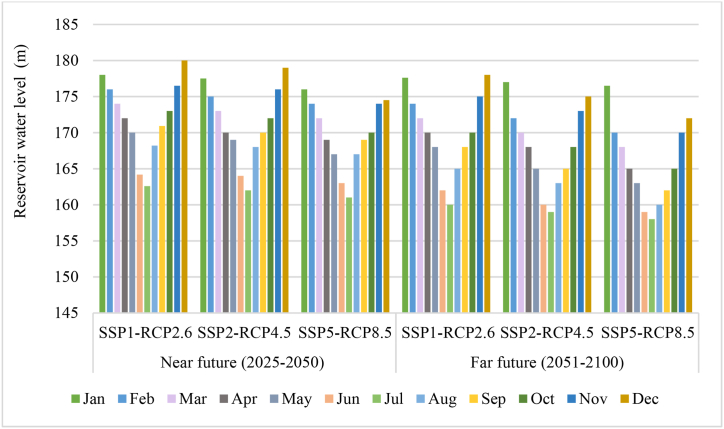
The average reservoir water level under climate and socio-economic changes.

Fig. (9) shows the average reservoir water level during two future time slices under SSPs-RCPs. The comparison shows that the maximum average storage level and the average reservoir level during the coming dry season are lower compared to the reference period. This indicates that the inflow intensity is very low during the storage period.

The results show that the water level in the reservoir varies according to the month and scenario. Generally, the water level is higher in winter (from December to February) and lower in summer (June to August). This could be due to seasonal patterns of precipitation and evaporation and dam performance in different seasons. The results also show that the water level in the reservoir tends to decrease as the release level increases.

In flood seasons, the reservoir's water level decreases compared to the water level of the reservoir in dry seasons, which is the reason for the dam's performance in flood control. Because the dam must release excess water to prevent flooding downstream. Of course, it is worth mentioning that this decrease in water level, like the input, has an increasing trend in the flood seasons, but it is less than in the dry season.

The highest decrease in water level is related to the pessimistic scenario in the distant time horizon during the flood seasons, while the lowest reduction in the water level is in the dry season, especially from December to February, under the pessimistic scenario in the distant time horizon.

The results show that the water level in the reservoir is sensitive to socio-economic and climate scenarios, and lower emissions and more sustainable development can help maintain or increase water availability in the reservoir. According to the SSP5-RCP8.5 scenario, the minimum average storage level will be slightly above 160 m by 2050, reaching a maximum of 175 m. Looking ahead to 2100, the SSP1-RCP2.6 scenario predicts a minimum average storage level of around 155 m, with a maximum of 175 m.

### 3.5 The hydropower potential

**Fig. 10:**
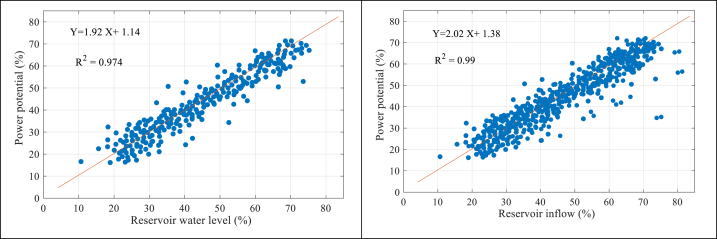
Correlation between a hydrological variable with power potential.

The evaluation of the correlation between the water level in the tank and the inflow of water has been conducted in this section, with a focus on the potential for generating hydropower. Hence, comprehending the influence of hydrological circumstances on the hydropower potential can enable the anticipation of the effects of socioeconomic and climate variations. The relationship between power potential and water reservoir level and inflow reservoir for The Gezhouba Dam is shown in Fig. (10).

The results show that the relationship between the water level of the reservoir and the inflow with the electricity potential is strong and direct, so that with the increase in the flow, the potential for electricity generation increases, and with the rise in the water level of the reservoir, the potential for electricity generation increases. Elite, according to the results obtained from the correlations, it is clear that the relationship between the input current and the electricity production potential is higher than the water level of the reservoir. Therefore, based on the results obtained, with the decrease in the water level of the reservoir and inflow to the reservoir, the potential for electricity has also been affected.

**Fig. 11:**
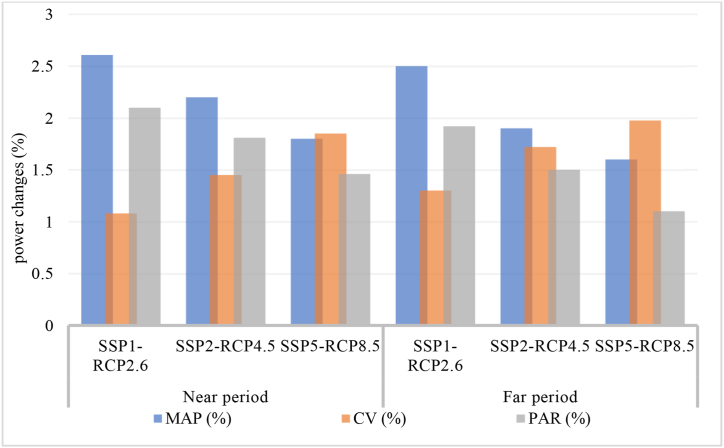
Power criteria changes under different scenarios.

Fig. (11) shows the predicted change of power criteria compared to the reference period under different scenarios.

The results show that by 2050, SSP1-RCP2.6 will have a CV of around 1.1 %, PAR of 2.1 %, and MAP of around 2.6 %. under SSP2-RCP4.5 will have a CV of around 1.3 %, PAR of 1.7 %, MAP of around 2.2 %. under SSP5-RCP8.5 will have a CV slightly above 1.4 %, PAR of 1.4 %, MAP of around 1.3 %.

By 2100, SSP1-RCP2.6 will have a CV of around 1.3 %, PAR of 1.8 %, and MAP of around 2 %. under SSP2-RCP4.5 will have a CV of around 1.6 %, PAR of 1.5 %, MAP of around 1.7 %. under SSP5-RCP8.5 will have a CV slightly above 2 %, PAR of 1.1 %, MAP of around 1.1 %.

The results show that the MAP of hydropower facilities decreases from the near term to the far term in all scenarios. This means that hydropower facilities face challenges in maintaining their output power in the long term due to the effects of climate change and socio-economic factors. According to the results, the minimum MAP under RCP8.5-SSP5 is 1.3 % and 1.1 % by 2050 and by 2100, respectively.

The results also show that the CV of hydropower plants increases from the near term to the far term in all scenarios. This means that hydropower facilities face more variability and uncertainty in their electricity production in the long term due to the effects of climate change and socio-economic factors. The maximum CV under RCP8.5-SSP5 is 1.4 % and 2 % by 2050 and 2100, respectively.

The results also show that the PAR of hydropower facilities decreases from the near-term to the far-off period in all scenarios. This means that in the long term, hydropower facilities face a greater risk of power shortages due to climate change and social and economic factors. The minimum PAR under RCP8.5-SSP5 is 1.4 % and 1.1 % by 2050 and by 2100, respectively.

**Fig. 12:**
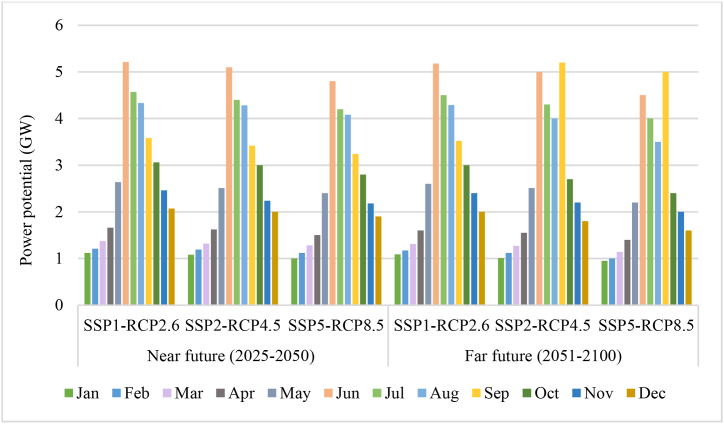
The average monthly power potential under climate and socio-economic scenarios.

Detailed information on seasonal changes and the performance of each measure in the near and far future is presented in Fig. (12).

The results illustrate that the hydropower plant's average monthly electricity production rates are at their highest during July, August, and June. Conversely, they are at their lowest in February, January, and December. This disparity can be attributed to the seasonal pattern of water flow, which tends to be higher in the summer months and lower during the winter.

Furthermore, when considering different scenarios, it is observed that the average monthly electricity generation rate is higher under the SSP1-RCP2.6 scenario, followed by the SSP2-RCP4.5 scenario. Conversely, it is lower under the SSP5-RCP8.5 scenario. Additionally, the results indicate that the average monthly electricity production rate of hydropower plants experiences a slight decrease from the near future to the far future. This decline can be attributed to the long-term impacts of climate change and socio-economic factors on various aspects such as water flow, water level differences, and dam performance.

Maximum Hydropower potential is observed from May to July in the SSP5-RCP8.5 scenario, reaching slightly over 5 GWh. In contrast, the highest Hydropower potential is recorded in January and December in the SSP1-RCP2.6 scenario, with a value approximately at or slightly below 1.1 GWh.

The study suggests various strategies to address the impacts of climate change on hydrological models and water resource management. Mitigation efforts focus on reducing greenhouse gas emissions, enhancing carbon sinks, and improving energy efficiency. Adaptation strategies include developing resilient infrastructure, implementing advanced forecasting systems, promoting efficient water use, diversifying water sources, and managing ecosystems. Dynamic management strategies are needed to adapt to changing inflow patterns and storage levels, optimizing water allocation for different uses. Hydropower facilities may need help with predicted decreases in water flow. Flexible operation protocols for dams are essential to balance flood control, water supply, and ecological needs. Policy recommendations involve integrating climate change into water management policies, investing in research, raising public awareness, and fostering international cooperation. These strategies aim to address climate change challenges and ensure sustainable water resource management and hydropower generation reliability.

## 4 Limitation and future research area

The study is constrained by various factors, such as the coarse resolution and higher equilibrium climate sensitivity of the CanESM5 model, potentially impacting the details of climate projections. Additionally, the SWAT model necessitates a significant amount of data, which may not be readily available in certain areas. While the SVR model's kernel function is vital for capturing intricate data patterns, accurately determining its hyperparameters can pose a challenge. Moreover, the LSTM model's computational cost is higher and more time-consuming to train compared to simpler models, with its sequential processing complicating parallelization efforts. Overcoming these limitations is crucial to gaining a transparent and comprehensive understanding of the models' restrictions, which is essential for precise result interpretation and guiding future research endeavors. The study seeks to offer an all-encompassing perspective on the models' limitations, a critical aspect for accurate interpretation and steering future research efforts.

Future research areas encompass investigating different renewable energy sources, examining the influence of new technologies on water conservation and energy efficiency, utilizing a more comprehensive array of climate models, performing an economic evaluation to comprehend the financial consequences of climate change on hydropower investments, and evaluating the possible effects of environmental and energy policies on the future functionality and sustainability of the dam. These factors can offer a thorough comprehension of the obstacles and possibilities confronting hydropower generation amidst global warming and industrial advancement, directing decision-makers and interested parties in making well-informed choices to guarantee the enduring sustainability of hydropower as a green energy option.

## 5 Conclusion

The Gezhouba Dam in China's Yangtze River is significantly impacted by climate change and socio-economic factors. The study uses an integrated methodology that combines Soil and Water Assessment Tools (SWAT) with Support Vector Regression (SVR) and Long Short-Term Memory (LSTM) models to predict inflow and reservoir levels. The Developed Thermal Exchange Optimizer is used to optimize the SWAT-SVR-LSTM model. The Canadian Earth System Model version 5 is used to assess changes in climatic variables across different socioeconomic routes and representative concentration pathways. The study found that climate change and socio-economic factors have led to increased warmth and alterations to the hydrological cycle, resulting in decreased water availability, reduced reservoir filling, and a decline in power production. The hydropower plant potential rates peak in July, August, and June, while their lowest levels occur in February, January, and December. The long-term consequences of climate change and socio-economic issues significantly affect water flow patterns and dam operational efficiency.

## Data availability statement

Research data are not shared.

## CRediT authorship contribution statement

**Chenyang Xiao:** Formal analysis, Data curation, Conceptualization. **Mohammad Mohammaditab:** Formal analysis, Data curation, Conceptualization.

## Declaration of competing interest

The authors declare that they have no known competing financial interests or personal relationships that could have appeared to influence the work reported in this paper.
